# Are the Sleep–Wake Cycle and Sleep Duration Ethnically Determined? A Comparison of Tibetan and Japanese Children’s Sleep

**DOI:** 10.3390/clockssleep6040046

**Published:** 2024-11-12

**Authors:** Ping Su, Masako Taniike, Yuko Ohno, Ikuko Mohri

**Affiliations:** 1 Department of Child Development, United Graduate School of Child Development, Osaka University, 2–2, Yamadaoka, Suita 5650871, Osaka, Japan; super05080605@qhnu.edu.cn (P.S.); masako@kokoro.med.osaka-u.ac.jp (M.T.); 2 School of Education, Qinghai Normal University, Xining 810016, China; 3 Department of Health Sciences, Osaka University Graduate School of Medicine, 1–7, Yamadaoka, Suita 5650871, Osaka, Japan; ohno@sahs.med.osaka-u.ac.jp

**Keywords:** infancy, sleep–wake rhythms, sleep duration, sleep-related habits, media use, environment

## Abstract

Background: Several environmental factors affect sleep. We investigated the sleep and sleep-related habits of preschool children living in Tibet and conducted an international comparison with those in Japan. Methods: We conducted a community-based cross-sectional study using the Chinese version of the Japanese Sleep Questionnaire for Preschoolers (JSQ-P-C) and compared the results with previous data on Japanese children. Results: The sleep status of 3113 children aged 3–6 years old in Qinghai province was evaluated. The average wake time and bedtime of the Tibetan children were 7:20 ± 0:31 and 21:16 ± 0:43, respectively. Their mean nocturnal sleep duration was 10.0 ± 0.7 h. In comparing 3-year-old children, the time for which they viewed TV in Tibet was shorter (65.5 ± 44.6 min) than that in Japan (149.7 ± 76.6 min), and the mother’s bedtime was earlier in Tibet (21:28 ± 2:14) than in Japan (23:20 ± 1:05). However, the bedtime and sleep duration of the Tibetan children (21:17 ± 0:37 and 10.0 ± 0.7 h) were fairly similar to those of the Japanese children (21:24 ± 1:57 and 9.8 ± 0.8 h). Conclusions: The late bedtime and short nocturnal sleep duration of Tibetan toddlers were the same as those of Japanese toddlers despite considerable differences in their lifestyle and environment.

## 1. Introduction

Recently, the importance of sleep to brain development has been reported. The pruning of excess synapses for learning and memory consolidation occurs during sleep [1,2,3]. Synaptogenesis and pruning are most active during early childhood [4]. European studies with large birth cohorts have suggested that sleep problems in infancy, such as a short sleep duration, frequent awakenings after sleep onset, late bedtimes, and irregularity of sleep patterns, are risk factors for high scores in attention deficit hyperactivity disorder and lower language acquisition at the ages of 5–6 years old, as well as depressive and psychotic symptoms in adolescence [5,6,7]. Getting enough and good-quality sleep is especially important in the early years of childhood.

Children who obtain sufficient sleep have better emotional and behavioral regulation [8,9]. It is not yet clear how much sleep each individual needs. The National Sleep Foundation (https://www.thensf.org (accessed on 24 Septemer 2024)) recommends that young children aged 3–5 years old sleep for at least 10 h per day. However, a short sleep duration has repeatedly been reported in Japanese infants and schoolers [10,11]. We also reported that Japanese children aged 3–6 years old have an average nighttime sleep duration of 9.8 h with 39.8% of these children go to bed after 22:00. In addition, we reported that children’s bedtimes were largely influenced by television viewing time and their caregivers’ lifestyles, including dinner time, bath time, and bedtime [11]. A short sleep duration has been reported for children in countries other than Japan. Some reports have revealed that Asian children, such as those in Taiwan, China, and the Republic of Korea, have later bedtimes and shorter sleep durations than children in Western countries [12,13,14]. Media and Internet use causes late bedtimes and short sleep durations in children [15]. In recent years, younger children in Japan have increasingly used media, with a 2019 survey by the Japanese Cabinet Office showing that more than 50% of 3-year-old children use the Internet [16].

Sleep is largely influenced by environmental factors such as lifestyle, sleep culture, co-sleeping, and sharing a room or bedding, which is common in many Asian countries. Japanese “futon” bedding facilitates movement to the parents’ “futon”. Bedtime resistance was more common in infants and preschoolers that co-slept or shared rooms [17]. Therefore, we developed the Japanese Sleep Questionnaire for Preschoolers (JSQ-P), which is adapted to Japanese sleep culture, enables the detection of sleep disorders and problematic sleep habits, and can screen for major sleep disorders, including obstructive sleep apnea, restless legs syndrome, and insomnia [18]. The JSQ-P consists of (1) demographic data such as bedtime, wake time, dinner time, TV watching time, and gaming time and (2) 39 items on sleep-related behaviors observed by the child’s caregivers, as previously described [19]. The JSQ-P has been used to study the sleep habits of Japanese preschool children [11], to assess sleep problems [8,20], and to conduct an international comparison between Malaysian and Japanese children [21]. Tibetan children also have a co-sleeping culture like Japan, but their environment is significantly different from Japan, with briefer TV viewing opportunities and a different lifestyle. Therefore, we assessed the sleep habits of Tibetan preschoolers using the Chinese version of the JSQ-P (JSQ-P-C). Its internal consistency and validity for Tibetan children were confirmed as previously described [22].

Qinghai province is located in the eastern hinterland of the Qinghai–Tibet Plateau in western China at 2000 m above sea level (masl). The main industries in Qinghai are sheep and yak farming. In 2016, Qinghai’s gross domestic product was USD 378.45 billion (7.6% of Japan’s), and its per capital income was USD 6.400 (16% of Japan’s). Electric appliances, such as mobile phones and video games, are less common among children in Qinghai than in Japan. Television (TV) programs for children are much less popular, with only one program, “QRTV-5”, compared to TV programs being available 24 h a day in Japan. As Tibet and Japan have the same sleeping habits of co-sleeping but very different environments in terms of media use, it was considered useful to study children’s sleep in both countries.

Using demographic data from the JSQ-P and JSQ-P-C, we compared the sleep and sleep-related habits of preschoolers in Tibet and Japan to determine whether sleep changes with the environment.

## 2. Results

### 2.1. Participants’ Characteristics

A total of 5244 questionnaires were distributed to kindergartens in four regions (Xining, Gonghe, Xinghai, and Maqin) in Qinghai province in China. A total of 3827 questionnaires were collected, with a response rate of 72.8%. The number of samples collected and the response rate in each area were 83.8% for 335 samples in Xining, 84.1% for 2088 samples in Gonghe, 83.2% for 1160 samples in Xinghai, and 25.0% for 244 samples in Maqin. Of the 3827 questionnaires, 180 were excluded due to blank responses, 458 due to missing data on age, and 76 due to missing data on sex. Ultimately, 3113 questionnaires were analyzed.

The mean age of the participants was 4.9 ± 0.4 years old, and 50.9% were boys. The ages of the fathers and mothers were 34.6 ± 6.1 and 32.3 ± 5.7 years old, respectively. Regarding the educational level of the caregivers, the ratio of caregivers with a junior high school education or lower was 49.0% in the fathers and 49.3% in the mothers. The proportion with a family income below CNY 4000 was 46.4% [23].

### 2.2. Sleep Habits and Nocturnal Sleep Duration of Children and Caregivers in Qinghai Province

In Qinghai, 91.7% of children sleep in the same room as their family members, and 76.9% sleep with their caregivers. Only 8.3% of the children slept alone in their rooms.

In all districts in Qinghai province, the median wake time of the children was 7:30, and 74.9% of the children woke between 7:00 and 7:20 (Table 1). Wake time increased in the 5-year-old children (*p* < 0.004). The median bedtime was 21:10; 64.7% of the children went to bed between 21:00 and 21:30, and 95% went to bed before 22:00. No significant differences were observed between the three groups. The median nocturnal sleep duration was 10.0 h in each age group. The median dinner time was 19:00 and was slightly earlier for the 3-year-old children (*p* < 0.001). The median TV viewing time was constant across all age groups. In conclusion, sleep-related daily schedules, such as wake time, bedtime, dinner time, and nocturnal sleep duration, were almost constant regardless of age.

TV viewing time was as short as 60 min in the Tibetan preschool children. A chi-square test was performed to analyze the effect of TV viewing time on sleep by dividing TV viewing time into two groups: less than 2 h and more than 2 h. In total, 1649 (80.6%) children who watched TV for less than 2 h went to bed before 22:00, and 397 (19.4%) went to bed after 22:00, whereas 409 (74.2%) children who watched TV for more than 2 h went to bed before 22:00, and 142 (25.8%) went to bed after 22:00. Thus, children who watched TV for more than 2 h were more likely to go to bed later (χ^2^ = 10.702, *p* < 0.001).

The average bedtime of the mothers in Qinghai was 21:28, and 63.4% of the parents went to bed between 21:00 and 22:00. Among the parents, 97.6% went to bed before 24:00. The average nocturnal sleep duration of the parents was 9.3 h, and 58.5% of them slept for more than 9 h.

### 2.3. Comparison of Sleep and Sleep-Related Schedules of 3-Year-Old Children in Qinghai Province and Japan

Table 2 shows a comparison of the sleep of 3-year-old children in Qinghai province and Japan. Their wake times were not significantly different, but their bedtime was significantly later in Japan than in Qinghai. Their nocturnal sleep duration was significantly shorter in Japan than in Qinghai. However, the differences in bedtime and nocturnal sleep duration are small, and the effect size is small (η^2^ 0.008 and 0.008, respectively). On the contrary, TV time and gaming time are significantly shorter in Tibet than in Japan (*p* < 0.001, η^2^ 0.135), but gaming time shows a small effect size (*p* = 0.001, η^2^ 0.002). The mother’s bedtime, which affects children’s sleep in particular in cases of co-sleeping, was significantly earlier in Tibet (21:28 ± 2:14) than in Japan (23:20 ± 1:05, *p* < 0.001, η^2^ 0.026).

## 3. Discussion

This study examined the sleep of children in Tibet, a region where TV and video games are less common, and compared the sleep of children in two regions, Tibet and Japan, which have similar sleep habits but very different environments, to investigate the effects of the environment on sleep.

### 3.1. Sleep Habits of Children in Qinghai Province

Similar to other areas of Asia, including Japan, most preschoolers in Tibet sleep in the same room as their caregivers [24,25]. According to the traditional concepts and lifestyles of child-rearing, caregivers in Tibet are sufficiently reliable observers of children’s sleeping conditions during the night to accurately assess the JSQ-P scores. In Qinghai province, the bedtime and nocturnal sleep duration were similar across age groups (Table 1). The duration spent watching TV and gaming remained constant with age. It has been reported that gaming time increases with age [23]; the gaming time was approximately 40 min/day for the 3- to 4-year-olds and 54 min/day for the 5- to 7-year-olds. Longer TV and gaming times have been reported to lead to later bedtimes and shorter sleep durations. In comparison, shorter TV and gaming times may promote consistent bedtimes and longer nocturnal sleep durations [26,27]. Previously, we reported that viewing more than 2 h of TV resulted in a bedtime after 22:00 [11]. Similarly, in Tibet, watching TV for more than two hours delays bedtime. As shown in Table 1, their gaming time is still short, and the TV watching time is not as long for preschool children in Qinghai as it is for those in Japan. Less time being spent watching TV in Qinghai may be because there are fewer TV programs for children in the evening. However, similar to other countries, TV and gaming times in Qinghai may increase in the future, which may affect sleep. This needs to be evaluated in follow-up studies.

### 3.2. Comparison of Sleep and Sleep-Related Daily Schedules in Qinghai Province and Japan

Several factors affect bedtime, including wake time, dinner time, TV viewing time, and gaming time [11,28]. As previously reported, parental lifestyle factors, such as parental bedtime, also affect children’s sleep [11,25]. In countries where co-sleeping is common, the mother’s bedtime has a significant influence on the child’s bedtime. Moreover, the Japanese way of bathing also causes a later bedtime. Japanese people typically bathe for approximately 30 min after dinner every day. In this study, the duration of time spent watching TV was less than half as long and the mother’s bedtime was approximately 2 h earlier in Tibet compared to in Japan (Table 2). Tibetans do not bathe as often due to their arid climate. Despite these favorable conditions for an early bedtime in Tibet, the actual bedtime of the Tibetan children was only 7 min earlier than that of the Japanese children.

The late bedtime of both Tibetan and Japanese children may be due to napping. Pal et al. reported that South Asian children had a shorter nighttime sleep duration and later sleep and wake times than white children, and nap duration was associated with nighttime sleep duration in white children but not in Asian children [29].

Reports have suggested that sleep duration and structure have substantial genetic components [30,31,32]. There was a significant interaction between sleep duration in children and 5-HTTLPR genotype polymorphism. Bedtime is associated with variants of the NPSR1 gene [33], and sleep duration is associated with MYRIP, PROK2, and a gene variant of ABCC9 [34]. Tibet is geographically distant from Japan, but its population shares more genetic similarities with Japan than other East Asian populations [35]. Genetic research has revealed that haplogroup D-M174 is predominantly distributed among Tibetans (40.96%) and Japanese (35.71%) but is less common or absent among other East Asian populations [35]. This suggests that Tibetans and Japanese share a close genetic relationship, despite living a long distance apart. Considering that Tibetan children also show late bedtime patterns despite not having busy schedules, like Japanese children, there may be a genetic relationship with bedtime.

Previous reports have highlighted differences in bedtime and nocturnal sleep duration between Asian and Caucasian countries [10,11,12,13,14]. In some Asian countries, the high population density and traffic congestion affect school attendance, and studying for exams until late at night may influence later bedtimes and shorter nocturnal sleep durations. In addition, studies that used questionnaires and actigraphy have suggested that genes may directly impact sleep quality by influencing certain sleep characteristics such as sleep duration, insomnia, and chronotype [36,37,38,39]. Further international comparisons are required to clarify the relative influences of genetic and environmental factors on sleep in children. Further investigation is warranted to define the distinctive criteria for good sleep habits among Asian children.

### 3.3. Limitations

This study had several limitations. There may have been parental and retrospective bias in the responses to the items because the parents completed the questionnaires based on recollection. As previously reported, each survey area’s sample size and response rate differed. The questionnaire used in this study was written in Han Chinese and may have been inaccessible to parents who only spoke Tibetan. This suggests the possibility of selection bias, which could explain the low response rates in some areas.

## 4. Participants and Methods

### 4.1. Participants

We surveyed kindergartens in four regions of Qinghai province: Xining City, Gonghe County, Xinghai County, and Maqin County. Table 3 shows the geographical characteristics, such as altitude, sunrise/sunset times, and population density.

The survey was administered to kindergartens, where the principals agreed to distribute the questionnaires in the classrooms. Questionnaires, such as the JSQ-P-C and questions on socioeconomic status, were distributed after explanation by the researchers (P.S. and I.M.). The caregivers of the children were informed that participation was free of charge; they were asked to complete the questionnaire anonymously and return it two days later. Submission of the questionnaire was considered consent to participation. The exclusion criteria were cases where consent could not be obtained and cases with a medical diagnosis of sleep disorders.

In the districts included in this study, almost all the children attended kindergarten schools. The travel time to school was approximately 30 min in Xining due to traffic congestion and approximately 15 min in the other areas. From Monday to Friday, the school day began at 8:30 and ended at 17:00. Each class consisted of 20–30 students. The fall and spring semesters ran from September 1 to mid-January and March 1 to late June, respectively. The survey was conducted between mid-September and mid-October 2019, a relatively stable period with fewer school events and community festivals.

### 4.2. Measurement

The JSQ-P (Supplemental Data S1) is a questionnaire developed to identify sleep-related lifestyles and common sleep problems in preschool-age children who have an Asian sleeping style, such as co-sleeping [18]. The JSQ-P was translated into Chinese and assessed through back-translation. The factor structure and reliability of the JSQ-P-C (Supplemental Data S2) were evaluated using exploratory factor analysis (EFA). Factors were extracted using principal component analysis, and varimax rotation was applied. Reliability was assessed using Cronbach’s α. The heterotrait–monotrait ratio of the correlations (HTMT) assessed the discriminant validity of each factor. The following eight subscales were obtained: “Insomnia”, “Restless legs syndrome (RLS)”, “Obstructive sleep apnea (OSA)”, “Excessive daytime sleepiness”, “Morning symptoms”, “Daytime behavior”, “Sleep deprivation”, and “Sleep habit”. The JSQ-P-C showed good internal consistency (Cronbach’s α = 0.917, 39 items), as described [22]. In the JSQ-P-C, we asked caregivers about their average wake time, bedtime, and sleep-related habits, such as dinner time, TV time, gaming time, and the mother’s bedtime in the past month.

### 4.3. Statistical Analysis

Fisher’s least significant differences test compared the sleep-related habits among three age groups of children, 3-year-olds, 4-year-olds, and children >5 years of age in Qinghai. To confirm whether there was an actual difference, H was calculated. For international comparison, we used data from Tibetan children (*n* = 312) and Japanese children (*n* = 2553) at the age of 3 years old, as previously reported [11], using the Mann–Whitney U test. Effect sizes (Cohen’s d) were calculated for the variables with significant differences.

## 5. Conclusions

A sleep survey of preschool children using a questionnaire was conducted in Tibet and compared with that conducted in Japan. For 3-year-old children, the amount of time spent watching TV and playing electronic games was lower in Tibet than in Japan. Regarding family lifestyle, which affects children’s sleep, mothers went to bed earlier in Tibet than in Japan. Still, there were no differences in the preschoolers’ bedtime and sleep duration between Tibet and Japan. It has been suggested that Tibetan and Japanese children sleep less than children in Western countries, probably because of genetic factors.

## Figures and Tables

**Table 1:** Differences in sleep-related habits by age in Qinghai province.

		Qinghai Province					*p*-Value
		Total *n* = 3113	3-y.o. *n* = 312	4-y.o. *n* = 928	≥5-y.o. *n* = 1873	H	
Wake time	Median	7:30	7:30	7:30	7:19	15.69	<0.004
	Interquartile range	7:00, 7:30	7:00, 7:30	7:00, 7:40	7:00, 7:30		
Bedtime	Median	21:10	21:30	21:10	21:20	3.00	0.22
	Interquartile range	21:00, 21:30	21:00, 21:40	21:00, 21:30	21:00, 9:30		
Nocturnal sleep duration (h)	Median	10.0	10.0	10.0	10.0	8.00	0.02
	Interquartile range	9.5, 10.5	9.5, 10.5	9.7, 10.5	9.5, 10.5		
Dinner time	Median	19:00	18:30	19:00	19:00	21.64	<0.001
	Interquartile range	18:30, 19:30	18:30, 19:00	18:30, 19:30	18:30, 19:30		
TV viewing time (min)	Median	60.0	60.0	60.0	60.0	1.47	0.48
	Interquartile range	30.0, 90.0	30.0, 90.0	30.0, 90.0	30.0, 90.0		
Gaming time (min)	Median	0.0	0.0	0.0	0.0	23.23	<0.001
	Interquartile range	0.0, 30.0	0.0, 0.0	0.0, 30.0	0.0, 30.0		

Median and interquartile range of sleep-related habits of children in Qinghai province. Fisher’s least significant differences test.

**Table 2:** Comparison of sleep and sleep-related habits of 3-year-old children in Qinghai province and Japan.

	Qinghai Province *n* = 312	Japan *n* = 2553	z	*p* Value	Effect Size (Cohen’s d)
Wake time	7:16 ± 0:24	7:21 ± 0:40	−0.301	0.764	0.034
Bedtime	21:17 ± 0:37	21:24 ± 1:57	−5.051	<0.001	0.597
Nocturnal sleep duration (h)	10.0 ± 0.7	9.8 ± 0.8	−4.863	<0.001	0.573
Dinner time	18:55 ± 0:47	18:42 ± 0:40	−3.337	0.001	0.385
TV viewing time (min)	65.5 ± 44.6	149.7 ± 76.6	−20.028	<0.001	1.281
Gaming time (min)	9.5 ± 25.1	12.6 ± 33.8	−3.276	0.001	0.377
Mother’s bedtime	21:28 ± 2:14	23:20 ± 1:05	−0.446	<0.001	0.051

Means and SDs of sleep time and sleep-related habits of children in Qinghai province and Japan. Mann–Whitney U test.

**Table 3:** Geographic data on the four districts of Qinghai province.

District	Altitude (m)	Sunrise (September 2019)	Sunset (September 2019)	Population (×10^3^) (Population Density/km^2^) *
Xining	2261	6:55	19:20	238.7 (323.0)
Gonghe	2862	7:00	19:25	13.4 (7.8)
Xinghai	3303	7:02	19:27	8.2 (5.6)
Maqin	3800	7:02	19:26	4.8 (3.7)

Geographic data on altitude, time of sunrise and sunset, and the population of each district. * China Statistical Yearbook (https://www.hongheiku.com/category/xianjirank/qhxsqrk (accessed on 8 May 2023).

## Data Availability

The data are unavailable due to privacy or ethical restrictions.
